# Novel pathomechanisms in inflammatory neuropathies

**DOI:** 10.1186/s12974-017-1001-8

**Published:** 2017-11-28

**Authors:** David Schafflick, Bernd C. Kieseier, Heinz Wiendl, Gerd Meyer zu Horste

**Affiliations:** 1 Department of Neurology, Westfälische Wilhems-University, Albert-Schweitzer-Campus 1, 48149 Münster, Germany; 2 Department of Neurology, Medical Faculty, Heinrich-Heine-University, Düsseldorf, Germany

**Keywords:** Experimental autoimmune neuritis, Guillain-Barré syndrome, Chronic inflammatory demyelinating polyneuropathy, Animal model, Th17 cells

## Abstract

Inflammatory neuropathies are rare autoimmune-mediated disorders affecting the peripheral nervous system. Considerable progress has recently been made in understanding pathomechanisms of these disorders which will be essential for developing novel diagnostic and therapeutic strategies in the future. Here, we summarize our current understanding of antigenic targets and the relevance of new immunological concepts for inflammatory neuropathies. In addition, we provide an overview of available animal models of acute and chronic variants and how new diagnostic tools such as magnetic resonance imaging and novel therapeutic candidates will benefit patients with inflammatory neuropathies in the future. This review thus illustrates the gap between pre-clinical and clinical findings and aims to outline future directions of development.

## Background

Inflammatory neuropathies are a heterogeneous group of autoimmune disorders affecting the peripheral nervous system (PNS) that can exhibit an acute or chronic disease course. They are rare, but cause significant and often permanent disability in many affected patients [1, 2]. Although our understanding of disease mechanisms in inflammatory neuropathies has improved in recent years, several important aspects remain poorly understood and have not yet been experimentally addressed. The current review aims to summarize available data regarding pathomechanisms in inflammatory neuropathies and covers findings both in animal models and human patients.

The Guillain-Barré syndrome (GBS) is the prototypic acute inflammatory neuropathy usually featuring a rapidly progressive, symmetrical, often ascending weakness of all extremities and variable sensory deficits [3]. In typical cases, initial symptoms can include pain, numbness, and paresthesia, followed by a rapidly progressive flaccid paresis of all limbs [4, 5]. Very severe impairments and potentially lethal secondary complications can develop. GBS has been reported to occur with an annual incidence ranging between 0.8 and 1.9 per 100,000 worldwide. Men are approximately 1.5-fold more frequently affected and the incidence increases with age [6]. Although the majority of patients recover, the prognosis can remain poor with severe disability or death in 9–17% of all GBS patients [7]. In many cases, GBS develops subsequently to minor infections in otherwise healthy individuals but is not associated with other autoimmune or systemic disorders. An infection-triggered cross-reactive auto-immune response is thought to be aberrantly directed against the peripheral nervous system [5]. This concept of infection triggered cross-reactivity is well established in axonal GBS and suspected in demyelinating GBS (see below). Intravenous immunoglobulins and plasma exchange are effective in GBS and numerous other pharmacological candidates have been suggested in animal models, but their bench-to-bedside transfer is lacking [8].

Chronic inflammatory demyelinating polyradiculoneuropathy (CIDP) is the most common chronic inflammatory neuropathy and is usually characterized by slowly progressive, symmetric, proximal, and distal paresis and sensory dysfunction [9]. Symptoms develop within two or more months and the course can be either chronically progressive or relapsing with stepwise progression [10]. Different variants and atypical courses have also been described [11]. The prevalence of CIDP greatly depends on the diagnostic criteria and ranges between 1 and 9 cases per 100,000 individuals with annual incidence rates at 0.50–1.60 per 100,000 [12–17]. Cerebrospinal fluid protein levels are elevated up to sixfold while other parameters are typically normal. Nerve biopsy shows features of demyelination and remyelination, onion bulb formation, nerve oedema, occasional epineurial, or endoneurial T cells [18–20], and macrophages scattered either throughout the endoneurium or in small perivascular clusters in the endoneurium [21, 22]. Glucocorticoids, plasma exchange, and intravenous immunoglobulins (IVIg) are effective in CIDP. The randomized controlled “intravenous immune globulin for the treatment of CIDP (ICE) trial” provided the highest level of evidence that IVIg reduces the progression of impairments in CIDP patients [23].

Both acute and chronic inflammatory neuropathies exhibit considerable heterogeneity and can be divided into different subtypes. Subtypes of GBS have been classified as acute inflammatory demyelinating polyradiculoneuropathy (AIDP), acute motor axonal neuropathy (AMAN), acute motor and sensory axonal neuropathy (AMSAN), and Miller Fisher syndrome (MFS) [24]. Chronic idiopathic axonal polyneuropathy (CIAP), multifocal motor neuropathy (MMN), and paraproteinaemic demyelinating neuropathy (PDN) have overlap with CIDP or can be considered as its variants [25]. Such diversity impedes the diagnosis, treatment, and development of new therapeutic interventions.

## The target antigen in GBS

Although the primary target antigen in GBS remains elusive, several recent studies have added interesting new candidates to the list of potential antigens [26]. Understanding the antigenic target could help to develop diagnostic or prognostic tests in the future and improve existing animal models. The hypothesis of microbial epitopes resembling endogenous peripheral nerve antigens, termed “molecular mimicry”, has been substantiated in the axonal subtypes of human GBS by several lines of evidence (reviewed in [27]). Briefly, there is a strong epidemiological association between GBS and preceding infections with *Campylobacter jejuni* (*C. jejuni*) and other infectious diseases [28]. Cell wall components of these infectious agents resemble endogenous lipids and may trigger a cross-reactive autoimmune response. Interestingly, a new infectious trigger for GBS was recently described.

Zika virus (ZIKV), a flavivirus such as yellow fever, dengue, Japanese encephalitis, and West Nile Virus, was recently identified as a novel trigger of GBS [29–32]. In one cohort, 98% of patients with GBS were tested positive for anti-ZIKV immunoglobulin M (IgM) or IgG. Most patients had a transient illness concordant with ZIKV infection several days before onset of GBS symptoms. The risk of GBS after ZIKV infection has therefore been estimated to be as high as 0.24/1000 infections, which is similar to the risk described for *C. jejuni* [33]. These data are supported by another study from Brazil during the recent major ZIKV outbreak. Between December 2015 and May 2016, evidence of recent ZIKV infection was found in serum and/or CSF of 77% of all GBS patients. Concurrently, GBS admission rates increased from an average of 1.0/month to 5.6/month [34]. First neurological symptoms were found to generally develop 6–10 days after symptoms of viral infection [33–35]. Only a minority of ZIKV-GBS patients had serological evidence of an autoimmune response against known GBS antigens [33]. Another study detected a considerable overlap between peptide sequences of ZIKV proteins and human structural myelin proteins [36]. These data support a molecular mimicry hypothesis in ZIKV-related GBS. Nerve biopsies show nerve fiber demyelination, axonal degeneration, and infiltration of mononuclear cells [37]. These findings raise concerns about a potential future health problem if endemic Zika virus infections could trigger clusters of GBS cases.

Autoantibodies binding components of the axonal membrane are found in a significant proportion of GBS cases [27]. Lipooligosaccharides from *C. jejuni* strains from a subset of axonal GBS patients carry ganglioside-like structures, resembling gangliosides enriched in axonal cell membranes (e.g., GM1, GD1a, GM1b, GalNAcGD1a) [28]. Therefore, the autoimmune reaction in axonal GBS is directed against axonal components. In support of this, some patients who received gangliosides as an experimental treatment for nonspecific pain syndromes subsequently developed an axonal GBS [38]. Recently, a novel glycoarray technique identified GM1, GA1, and GQ1b IgG antibodies in a high number of GBS patients and GQ1b antibodies were preferentially enriched in GBS patients with ophthalmoplegia [39]. In addition, some GBS sera were shown to strongly and specifically bind monoaminergic neurons in rat brain suggesting a potential interference of auto-antibodies with ion channels and neuronal receptors in GBS. This could explain neuropsychiatric and autonomic abnormalities in GBS [40].

Animal models for studying axonal GBS and anti-ganglioside antibodies in GBS have been described by repeatedly immunizing rabbits against axonal gangliosides GD1b (in ataxic sensory neuropathy) and GM1 (in acute motor axonal neuropathy) [41], causing axonal experimental allergic neuritis (EAN) with flaccid paresis [42], axonal damage, and ganglioside-directed antibody responses [43]. Some anti-ganglioside antibody-mediated neuropathies are characterized by a disruption of paranodal junctions and ion-channels at the nodes of Ranvier [44]. Ganglioside antibodies were speculated to cause a transient and initially reversible disruption of axonal impulse propagation. Only if secondary axonal degeneration ensues, disability will be permanent. A novel category of nodo-paranodopathies was therefore proposed for neuropathies associated with anti-ganglioside antibodies targeting nodal regions [45]. The applicability of this classification to standard clinical care remains to be determined.

Mice lacking complex gangliosides develop exaggerated humoral responses to gangliosides when immunized with *C. jejuni*, lipooligosaccharides [46], or gangliosides [47], indicating that tolerance-inducing mechanisms exist if endogenous gangliosides are present. In addition, passive transfer of ganglioside-directed antibodies induced axonal pathology in recipient animals [48], although sera from GBS patients did not exacerbate adoptive transfer EAN [49]. The available evidence thus supports that axonal GBS and corresponding ganglioside-induced animal models share a common antigenic target and disease mechanism.

The availability of animal models contrasts with the lack of novel therapeutic strategies in axonal GBS.

The target cellular structure or defined antigen in demyelinating human GBS remains unknown. Several studies have reported auto-antibodies or a cellular immune reaction against myelin proteins although this only holds true in a small proportion of demyelinating GBS patients [50–53]. Although mostly associated with axonal subtypes, lipids may also become target antigens in some demyelinating GBS cases [54]. Although myelin proteins can be used to elicit animal models of GBS (see below), no single antigenic target has been confirmed in a greater proportion of demyelinating GBS patients and no corresponding microbial structure with myelin-like properties has been identified [28]. Of note, a plethora of therapeutic strategies have been tested in demyelinating GBS models although the model fails to mirror the antigen specificity of the human disease [8]. Polymorphisms in the genes encoding interleukin 17 (IL-17) and intercellular adhesion molecule 1 (ICAM-1) lead to higher expression and may predispose to developing GBS [55]. Of note, both IL-17 and ICAM-1 have been associated with inflammatory neuropathies in animal models [56, 57].

## Recent advances in understanding CIDP

Chronic inflammatory neuropathies such as CIDP lack strong evidence for a molecular mimicry-like mechanism described in GBS. The chronic and delayed onset of symptoms may potentially preclude the identification of triggering agents. CIDP can occur in autoimmune-susceptible patients with some patients also exhibiting inflammatory demyelination of the central nervous system with patchy regions of demyelination and oedema and inflammatory infiltrates seen in histology and magnetic resonance imaging [58]. When considering potential antigens, it is important to be aware that the majority of myelin proteins are specifically present in either central or peripheral nervous system but not both. Antibody responses against the myelin proteins P0 and P2 [59] and peripheral myelin protein 22 (PMP22) have been reported [50, 60], while other studies have not confirmed antibodies recognizing myelin I proteins in a relevant proportion of patients [53, 61]. Anti-ganglioside antibodies can be detected in some CIDP patients [62]. In addition, T cell reactivity against myelin proteins may occur in CIDP patients [51]. Thus, proteins of compact myelin can become target of chronic autoimmunity in some CIDP patients.

Several studies have interestingly identified the axonal node and paranode as novel potential targets of autoimmunity in chronic (and acute) inflammatory neuropathies [63]. Recent studies identified IgG4 antibodies against Neurofascin 155 (NF155) in a comparably homogenous group of CIDP patients. Patients with these antibodies were younger at onset, presented sensory ataxia, a disabling and characteristic tremor, and CNS (central nervous system) demyelination, and had a very poor response to IVIg treatment [64, 65]. A recent study also showed that immunoglobulins recognizing NF140/186 may cause reversible conduction block and demyelination which does respond to IVIg treatment. These findings suggest a complement-independent mechanism of NF140/186 IgGs [66]. Antibodies to Neurofascin have also been reported in EAN and as a potential target antigen in multiple sclerosis [67, 68]. Paranodal contactin-1 was identified as an additional potential antigenic target [69, 70]. Schwann cells exposed to sera from CIDP patients may be less effective at supporting growth of regenerating axons [71]. These observations indicate that different paranodal components can become targets of the immune response in CIDP and that the mechanisms leading to axonal degeneration may vary. Such novel antigenic targets may also reinvigorate the search for corresponding microbial antigens and molecular mimicry-like mechanisms.

Several studies have reported the endemic occurrence of a chronic inflammatory neuropathy in abattoir workers exposed to porcine brain tissue aerosols [72]. These cases provide interesting proof-of-principle, that active immunization against nervous system tissue by aerosol exposure can cause chronic inflammatory neuropathies in humans and argue for an antigenic target shared between central and peripheral nervous systems.

Primary structure and complementarity-determining region (CDR) 3 spectratyping of the T cell receptor (TCR) repertoire in CIDP patients has indicated that CD8^+^ T cells exhibit a much broader activation than CD4^+^ T cells and that IVIg preferentially ameliorates this. CD8^+^ T cells may thus have a crucial role in the immunopathogenesis of CIDP [73]. In accordance to these investigations, biopsies from CIDP patients showed a T cell receptor repertoire which has a strong monoclonal and oligoclonal restriction. Overlaps between CD8^+^ T cell clones in biopsies and blood have been observed, indicating an antigen-driven, major histocompatibility complex I restricted attack of peripheral nerve tissue components by CD8^+^ T cells [74]. Because of its CD8^+^ T cell-mediated autoimmune peripheral neuropathy, the L31 mouse strain (discussed in detail below) might be the appropriate model for this aspect [75]. Overall, several interesting new observations have been made in CIDP, but the antigenic specificity and the exact mechanisms of the autoimmune response in CIDP require future study.

## Animal models of acute demyelinating inflammatory neuropathies

Animal models allow deciphering pathomechanisms of human disorders and enable preclinical testing of potential future treatments. The first available animal model of inflammatory peripheral neuropathies was generated by immunizing a susceptible rabbit strain with PNS myelin emulsified in complete Freund’s adjuvant (CFA) [76]. The disease resulting from such an induction protocol was named experimental autoimmune (initially “allergic”) neuritis and was later extended to various other animal species and strains including rats, mice, monkeys, and guinea pigs (Table 1). The Lewis rat strain enables most reliable EAN induction and has become a widely used GBS model. EAN induction in mice has proven to be problematic (see below).

**Table 1:** Animal models of inflammatory neuropathies including references

Animal model	Induction	Possible antigens	Description	Ref.
Rat				
Lewis	Active	PNS myelin, P0(180–199), P2(53–78), P2(57–81)	Frequently used EAN models	rev. in [208] e.g., [209–211]
	Active	PMP22	Mild course EAN	[78]
	Active	PNS myelin + Cyclosporine A	CIDP-like chronic relapsing, not robust	[86]
	Adoptive transfer	P0, P2, P0(180–199), P2(61–70), MAG	Rapid onset EAN, CIDP-like relapsing if transferred repeatedly	rev. in [94, 208]
BN, SD, BUF, Wistar	Active	PNS myelin	Less-severe EAN course	[212]
Dark Agouti	Active	PNS myelin	CIDP-like relapsing	[85]
Mouse				
C57/B6	Active	P0(180–199), P0(106–125) + PTx	Varying effectiveness reported	[213, 214]
IL10 over-expressing C57/B6	Spontaneous	–	CIDP-like, demyelination, muscle weakness, paralysis, axonal loss	[97]
SJL	Active	P2	Mild course EAN	[215]
		Myelin + PTx (+ IL-12)	Severe course EAN	[216]
Balb/C	Adoptive transfer	Myelin basic protein	Peripheral and central demyelination	[217]
NOD	Active	*Oral C. jejuni*	Mild course acute motor axonal neuropathy (AMAN)	[83]
IL-10^−/−^ NOD	Active	*Oral C. jejuni*	Acute motor axonal neuropathy (AMAN)	[83]
B7-2^−/−^ NOD	Spontaneous	–	Spontaneous autoimmune neuropathy CIDP-like	[99]
	Active	*Oral C. jejuni*	Acute inflammatory demyelinating polyneuropathy (AIDP)	[83]
ICAM^−/−^ NOD	Spontaneous	–	Spontaneous autoimmune neuritis	[107]
PD-1^−/−^ + H-2^b^ NOD	Spontaneous		Autoimmune peripheral neuropathy	[110]
IL-2 mAb depleted NOD	Spontaneous		Autoimmune peripheral neuropathy	[106]
Aire ^GW/+^ NOD	Spontaneous		Autoimmune peripheral neuropathy, CIDP-like	[112]
ICOS/ICOS-L^−/−^ NOD	Spontaneous		Multifocal autoimmune neuropathy	[111]
Other				
Guinea pig	Active	PNS myelin		[218]
Monkey	Active	PNS myelin, P2		[219]

*Abbreviations*: *AIDP* acute inflammatory demyelinating polyneuropathy, *BN* Brown Norway rat, BUF Buffalo rat, *CIDP* chronic inflammatory demyelinating polyradiculoneuropathy, *EAN* experimental autoimmune neuritis, *GBS* Guillain-Barré syndrome, *IL* Interleukin, *NOD* non-obese diabetic, *P0* myelin protein zero, *P2* myelin protein, *PMP* peripheral myelin protein, *PNS* peripheral nervous system, *PTx* pertussis toxin, *Ref* reference, *rev* reviewed, *SD* Sprague Dawley rat, *SJL* Swiss Jim Lambert mouse

**Fig. 1:**
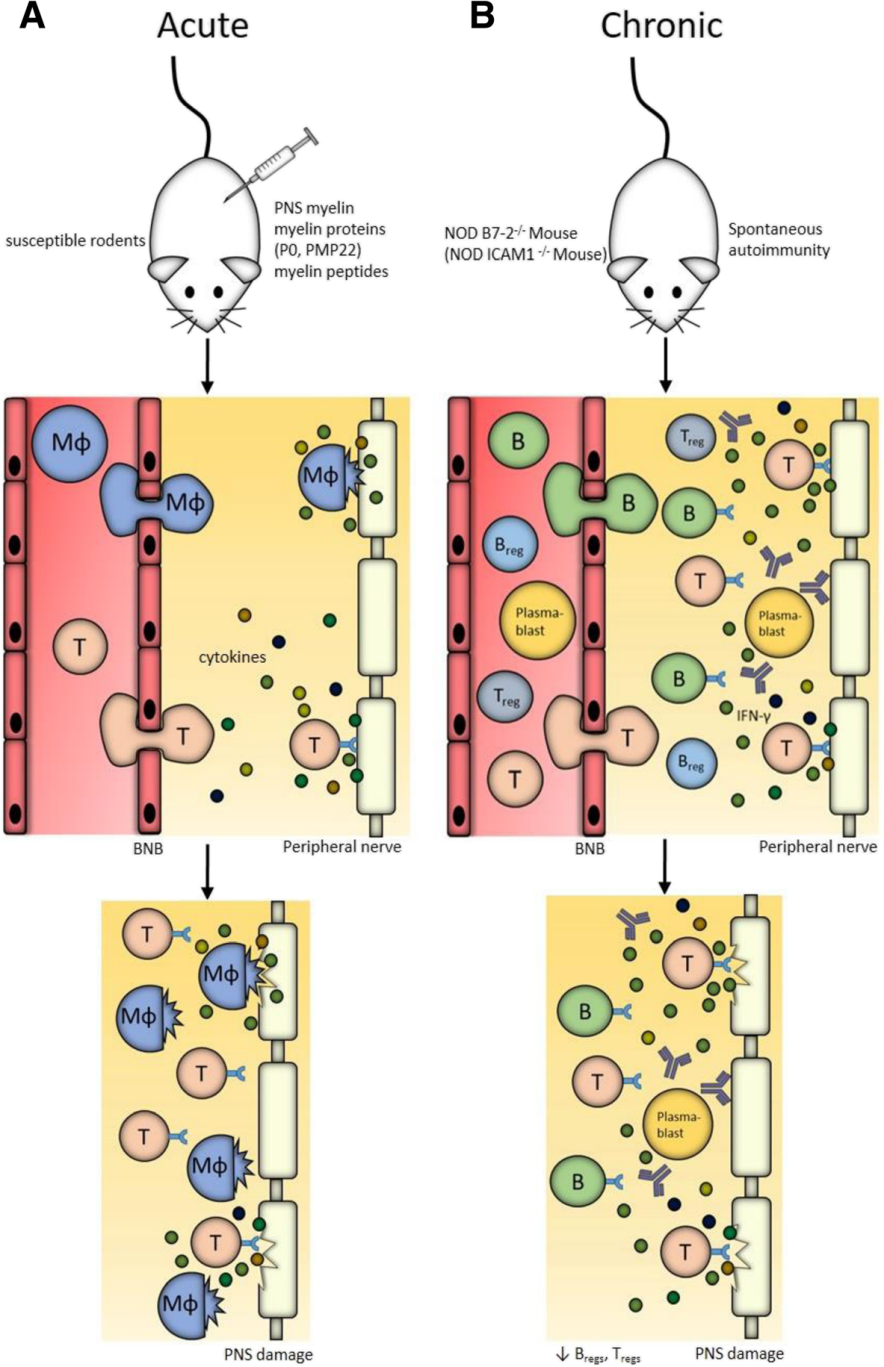
Representative animal models for GBS, CIDP, and underlying mechanisms. **a** Experimental autoimmune neuritis (EAN) is the animal model of acute peripheral neuropathies such as GBS. Induction is achieved by immunizing susceptible rodents (e.g., Lewis rats) with PNS myelin, peripheral myelin homogenates, myelin proteins (P0 or PMP22) or myelin-derived peptides. Immunization leads to a myelin-directed T cell response characterized by T cell and macrophage infiltration. Cytokine production mediates peripheral nerve damage and attraction of further inflammatory cells. **b** NOD B7-2^−/−^ and NOD ICAM1^−/−^ mice develop spontaneous CD4^+^ T cell-mediated neuritis with parallels to CIDP in humans. The neuritis is characterized by P0-specific CD4^+^ T cells that infiltrate into the PNS, IFN-γ dependency, an increased B cell reactivity to P0, expansion of P0-specific plasmablasts and lower frequencies of T_regs_/B_regs._ In the case of NOD ICAM1^−/−^ mice, mechanisms are slightly different than depicted here. In this case, the neuritis is characterized by enhanced IL-17 production, macrophage and B cell infiltration without changes in T_reg_ levels

Different components of PNS myelin can be used to trigger EAN. Lewis rats can be immunized with peripheral myelin homogenates, myelin proteins, or myelin protein-derived peptides to develop EAN (Fig. 1). The most abundant structural myelin protein is myelin protein zero (Mpz or P0) and can be used to induce EAN [77]. Less-severe forms of rat EAN are triggered by immunization against peripheral myelin protein of 22 kDa (PMP22) [78]. In addition to actively induced EAN, transfer of stimulated T lymphocytes that are reactive against various myelin antigens, including myelin proteins P2, P0, and derived peptides, evokes adoptive transfer EAN (AT-EAN) in receiving host animals (Table 1). These adoptive transfer studies have identified myelin-associated glycoprotein as another potential target in EAN [79]. Collectively, these studies have established the heterogeneity of potential antigenic targets in the PNS and allow understanding of general principles of PNS inflammation.

Actively induced EAN generates a PNS myelin-directed T cell response that triggers an acute monophasic cellular immune reaction within peripheral nerves [80]. Several aspects of human GBS are resembled in such actively induced EAN. Histologically, macrophages and T cells are the predominant infiltrating cells and cytokines mediate local tissue damage in the PNS and amplify the immune response by further attracting inflammatory cells [8, 80, 81]. Like EAN, GBS features an acute onset and monophasic course of disease that is triggered by exposure to proteins or microbial epitopes resembling self, respectively. Myelin protein autoreactivity, however, has been described in a small proportion of GBS patients (see above). Infiltration of mononuclear cells and immune cell-mediated myelin destruction in the PNS occurs at least in a proportion of GBS patients with a demyelinating disease phenotype [82]. Myelin component-induced EAN is thus a useful model of demyelinating GBS but fails to reproduce several aspects of the human disease.

Other evidence argues against the adequacy of myelin protein-induced EAN in reflecting GBS: A myelin directed autoimmune response is absent in most GBS patients [26, 52] and an evidence for a microbial epitope, resembling any human PNS myelin structure, is lacking. Furthermore, a long list of treatments was found effective in EAN but has not resulted in the establishment of novel therapeutic options in GBS (reviewed in [8]).

Of note, a recent publication described induction of a GBS-like disease in non-obese diabetic (NOD) mice after oral infection with *C. jejuni* strains from GBS patients. Mice developed autoantibodies against GM1, GQ1b, and GD1a gangliosides, inflammatory peripheral nerve lesions, axonal damage, and clinical impairments while antibiotic treatment exacerbated the symptoms. The authors also tested different NOD mutants and found that *C. jejuni* infection triggered T cell-dominated AIDP-like disease in the absence of B7-2 and axonal AMAN-like disease in the absence of IL-10. Therefore, NOD mice infected with *C. jejuni* may serve as a feasible novel GBS animal model enabling distinct disease phenotypes depending on host genotypes [83]. This tool appears especially suited to dissect intestinal triggers of PNS autoimmunity.

A recent publication using a rabbit model of axonal GBS demonstrated that the immunoglobulin G-degrading enzyme of *Streptococcus pyogenes* (IdeS) which cleaves IgG antibodies into fragments reduces complement-mediated axonal degeneration in anterior spinal roots. This is paralleled by a reduced disruption of voltage-dependent sodium (Nav) channels and less activated C3 at the nodes of Ranvier [84]. These data indicate that IdeS might be a promising therapeutic strategy for GBS.

## Animal models of chronic inflammatory neuropathies

Animal models of CIDP or other chronic inflammatory neuropathies are less well established than acute models. Active immunization paradigms to induce chronic PNS inflammation have been reported: Rats of the Dark Agouti strain develop relapsing PNS inflammation and active myelin basic protein (MBP) immunization together with Cyclosporine A (CsA) treatment in Lewis rats, triggers a relapsing disease course with some CIDP-like features [85, 86]. Administration of low-dose CsA induces a relapsing or chronic persisting EAN course [86, 87]. The mechanisms for this chronification are still unknown but altered production of cytokines may be one potential explanation [88, 89]. IL-17A production was significantly enhanced after CsA cessation in relapsing murine EAE [90]. These data suggest a role of IL-17A in the relapses induced by CsA. One may also speculate that CsA suppresses T_reg_ development by reducing IL-2 production [88, 89]. In fact, IL-2 is required for T_reg_ proliferation, survival, and activity and inhibition of IL-2-dependent T_reg_ proliferation together with increased IL-17A production due to CsA could trigger relapses [91–93].

Repeatedly performing adoptive transfer EAN may also reflect recurrent attacks in CIDP [94]. These induced models of CIDP, however, do not phenocopy the chronic progressive nature of CIDP. A number of studies have added several new animal models of chronic inflammatory neuropathies:

First, the so-called L31 mouse strain is characterized by constitutive expression of the costimulatory B7-2 (CD86) molecule on antigen-presenting cells. These mice develop spontaneous demyelination and immune cell infiltration of the peripheral nerves [95]. Depletion of CD4^+^ T cells in L31 mice expanded CD8^+^ T cells and accelerated and exacerbated the disease. CD8^+^ T cells are thus considered key drivers of the disease [96]. These mice exhibit deficits in motor and sensory functions with acute onset and continuous progression [75].

Second, mice on C57BL/6 background, transgenically expressing IL-10 under the control of the promotor of the vitelliform macular dystrophy 2 (VMD2) gene mutated in macular degeneration, spontaneously develop neuritis within 10 to 20 weeks of age. This neuritis is one of the very rare examples of a “pro-inflammatory function” of generally anti-inflammatory IL-10 and is caused by an IL-10-dependent ICAM-1 upregulation which triggers macrophage influx [97].

Third, several novel models of CIDP, based on the NOD mouse strain, have been established. The NOD mouse is prone to developing spontaneous autoimmunity such as a type 1 diabetes-like phenotype [98]. Initially, Salomon et al. reported in 2001 that NOD mice deficient in the costimulatory molecule B7-2 are protected from diabetes but instead spontaneously develop a CD4^+^ T cell-mediated, slowly progressive demyelinating peripheral neuritis predominantly in female (Fig. 1) [99]. Later studies found the spontaneous neuritis to be dependent on interferon γ (IFN-γ) and on a T-helper 1 skew in these mice [100]. The P0_180–199_ epitope of myelin protein zero was identified as a potential antigenic target, and a neuritogenic T cell receptor transgenic mouse line [101], recognizing the P0_1–25_ epitope, was generated from this mouse strain [102]. In addition, an increased B cell reactivity to myelin protein zero (P0) and expansion of P0-specific plasmablasts was observed, while depletion of B cells with an anti-CD19 antibody led to decreased disease severity [103]. Another therapeutic option is FTY720, also termed Fingolimod, which was shown to intermit the progression of chronic neuritis in B7-2-deficient NOD mice by either depletion of T cells or indirect inhibition of pathogenic T cell expansion [104]. A recent study demonstrated lower frequencies and numbers of regulatory T cells (T_regs_) and regulatory B cells (B_regs_) in this mouse line and found these regulatory cell types to suppress spontaneous autoimmunity [105]. Summarizing, the B7-2-deficient NOD mouse mirrors the slowly and chronic progressive nature of CIDP, does not require immunization, and has been successfully used to study mechanisms and test several novel therapies of CIDP.

Fourth, spontaneous autoimmune neuritis has also been reported in other mouse strains on the NOD genetic background. Antibody-mediated depletion of IL-2, required for the maintenance of T_reg_, caused spontaneous neuritis in NOD mice and antibody-mediated depletion of regulatory T cells exacerbated chronic neuritis [106, 107]. NOD mice carrying deficiency in programmed cell death 1 (PD-1)—an adhesion molecule with costimulatory function—develop diabetes with increased severity [108], while PD-1-deficient NOD mice, carrying the anti-diabetogenic major histocompatibility complex (MHC) haplotype H-2^b^, are protected from diabetes but develop spontaneous peripheral neuritis [109, 110]. NOD mice deficient in the inducible costimulator (ICOS) and ICOS ligand (ICOS-L) spontaneously develop multifocal autoimmunity in the nervous system and in muscle tissues including spontaneous neuritis [111]. NOD mice with a partial loss of *Aire* gene function (NOD.Aire^GW/+^) spontaneously develop a peripheral nervous system autoimmunity comparable to CIDP. In these mice, a partial loss of *Aire* gene function leads to reduced P0 expression in the thymus resulting in an escape of P0 self-antigen-recognizing T cells from negative selection and thus central tolerance [112].

In addition, a recent study reported a spontaneous chronic neuritis in ICAM-1-deficient NOD mice. Interestingly, this neuritis is CD4^+^ T cell-dependent featuring enhanced production of pro-inflammatory IL-17 without any alternation in T_reg_ numbers. T cells as well as macrophages and B cells infiltrate into peripheral nerves and show autoreactivity against P0 and an altered TCR repertoire. Thymus transplantation experiments identified an altered thymic selection of T cells rather than altered costimulation of T cells in the periphery to underlie the disease [56]. Altered costimulation may thus affect the thymic selection process and generally shift autoimmunity to peripheral nerves. It remains to be determined why the murine peripheral nervous system is preferentially prone to become an autoimmune target in specific pro-inflammatory settings and how this knowledge may be exploitable for human patients.

## T cell subtypes in inflammatory neuropathies

The expansion of knowledge regarding CNS autoimmunity has not been paralleled by studies in the PNS. In particular, many novel subsets of CD4^+^ T cells have not been studied in inflammatory neuropathies at all.

For many years, attention focused on Th1 and Th2 helper T cells—two different differentiation programs of naïve CD4^+^ T cells. This long-accepted dichotomous model has been extended by the description of pro-inflammatory Th17 producing the cytokines IL-17A, IL-17F, and IL-22 [113]. Under physiological conditions, Th17 cells clear fungal pathogens. However, Th17 cells are also potent inducers of tissue inflammation and have been associated with many experimental and human autoimmune conditions [114]. The contribution of Th17 cells in the pathogenesis of inflammatory neuropathies in rodents and in humans has been poorly defined.

The presence of IL-17-producing cells has been described in murine EAN and recombinant IL-17 deteriorated the acute phase of rat EAN [115]. Interestingly, experimental treatments with Atorvastatin and Fingolimod reduced the accumulation of IL-17-producing cells in the PNS of experimental animals [116, 117].

In human CIDP patients, the percentage of peripheral Th17 cells and IL-17 plasma levels were increased specifically in patients with active disease [118]. The concentration of IL-17 and IL-6 (which induces IL-17) were increased in cerebrospinal fluid of CIDP patients [119], while another study reported an increased IL-17 production by blood mononuclear cells, although this was not specific for this cytokine [120]. In accordance, we recently found an increased number of IL-17-producing cells, preferentially in sural nerve biopsy sections of CIDP patients with high disease activity. The number of IL-17-producing T cells, before IVIg treatment, correlated with the beneficial response in ICAM-1^−/−^NOD mice. A high number of IL-17-producing cells thus predicted a good response to IVIg treatment while surprisingly IL-17 production in secondary lymphoid organs was unchanged [57]. These observations indicate IL-17 as a prognostic marker of disease activity and the efficacy of IVIg treatment.

In GBS patients, the frequency of circulating Th1, Th17, and Th22 cells was significantly increased and plasma and CSF (cerebrospinal fluid) levels of IL-17 and IL-22 were also elevated during the acute disease stage. The levels of both cytokines were directly correlated with the disability scale scores of the patients [121]. IVIg therapy downregulated these cells and respective cytokines [122], indicating a potential pathogenic role of Th17 cells in GBS. Different Th17 cell-inhibiting compounds have been described in experimental autoimmune encephalomyelitis (EAE) models [123, 124]. One may speculate that such findings could be transferable to inflammatory peripheral neuropathies and open new therapeutic avenues in the future.

T_reg_ cells expressing the transcription factor forkhead box P3 (FoxP3) have been studied extensively and deficiency of T_regs_ causes a severe and lethal autoimmune syndrome in humans (termed IPEX, immunodysregulation polyendocrinopathy enteropathy X-linked syndrome) and mice [125, 126]. T_reg_ dysfunction has been implicated in various human inflammatory disorders (reviewed in [127]). An impaired function or reduced numbers of T_regs_ have also been described in CIDP and GBS patients [128, 129]. Experimental studies have confirmed the presence of T_regs_ in EAN, and the beneficial effect of treatments in EAN has been attributed to increased T_reg_ numbers [115, 130]. In EAN, T_reg_ depletion with antibodies increased the clinical severity, the proliferation of myelin-specific T cells, and peripheral nerve inflammation in the initial priming phase of inflammation. Notably, systemic autoimmunity caused by lack of FoxpP3^+^ T_regs_ does *not* manifest with neuritis [131]. Lack of T_regs_ thus does not precipitate PNS-specific autoimmunity but exacerbates ongoing PNS inflammation triggered by other mechanisms.

IVIg treatment in GBS patients expanded T_reg_ cells [132]. Protocols for the in vitro induction of T_regs_ from naïve CD4^+^ T cells of murine and human origin have been developed [133]. It is intriguing to speculate whether specific induction of stable T_regs_ would ameliorate EAN and pose a potential future treatment of GBS or CIDP. Another option is the suppression of leukocyte trafficking to the PNS. Preconditioning rats with forced exercise decreased EAN severity due to altered leukocyte composition and egress of autoreactive Th1 cells from draining lymph nodes [134]. These data argue for an immunosuppressive and beneficial effect of exercise in inflammatory neuropathies. In a recent study, treatment of EAN mice with PD-1 ligand (PD-L1) inhibited T cell proliferation and shifted T cell responses from a Th1/Th17 to a Th2/T_reg_ phenotype [135]. PD-L1 may thus constitute a potential future treatment option.

## Leukocyte trafficking across the blood-nerve barrier in GBS and CIDP

Trafficking of leukocytes across the blood-nerve barrier (BNB) is an important step in inflammatory neuropathies such as GBS and CIDP. Several different factors like selectins (E- and P-Selectin), chemokines and chemokine receptors, adhesion molecules, and integrins play a role in the process [136]. No animal model allows investigating trafficking of leukocytes at the BNB in real time in vivo. Therefore, different in vitro models using static and flow conditions have been established.

In vitro studies using primary endoneurial endothelial cells from human sciatic nerves showed that activation of endoneurial endothelial cells by the chemokines TNF-α and IFN-γ leads to induction of pro-inflammatory chemokines such as CCL2, CXCL9, CXCL11, and CCL20. The observed upregulation of CCL2 is thought to play a major role in mediating monocyte/macrophage trafficking through interaction with CCR2 in humans [137]. Studies on a mouse model of peripheral neuropathic pain support the importance of CCL2/CCR2 in monocyte/macrophage infiltration into peripheral nerves. Upregulation of CCL2 correlated with local monocyte/macrophage infiltration while sciatic intraneural microinjection of CCL2 lead to a recruitment of these immune cells [138]. Furthermore, in humans, CXCL2-3 and CXCL8 are thought to drive neutrophil trafficking through interaction with chemokine receptors CXCR1 and CXCR2. It was also demonstrated that the activation state of endothelial cells is more important determining cellular trafficking across the BNB than the activation state of leukocytes [137].

A multi-step paradigm for leukocyte trafficking at the BNB was proposed in parallel to leukocyte trafficking in other tissues. The initial step is the rolling of leukocytes on the endothelial monolayer surface, followed by arrest and firm adhesion at intercellular membranes and some transmigration via the paracellular route presumably at places with high chemokine presentation. Leukocytes extracted from AIDP patients showed an α_M_ integrin-ICAM-1-dependent adherence to the BNB in vitro with CD14^+^ monocytes being the most prevalent subpopulation [137]. In fact, α_M_ integrin (CD11b) was found in sural nerve biopsies of AIDP patients in large clusters of endoneurial CD11b^+^ leukocytes associated with demyelinating axons. These observations were paralleled in a murine EAN model with significant symptom amelioration using a monoclonal antibody against CD11b [139].

ICAM-1 blocking antibodies prevented or suppressed the development of EAN in the induction phase of disease rather than in the effector phase, indicating the importance of ICAM-1 in BNB trafficking [140, 141]. Other studies in murine EAN revealed that CCL2-CCR2 and CXCL10-CXCR3 have a direct effect on leukocyte transmigration to the peripheral nerve. Both receptors and ligands accumulate in the sciatic nerves of EAN mice [142]. Similar observations were made in sural nerve biopsies from GBS, AIDP and CIDP patients. Specific chemokine receptors are expressed by endoneurial macrophages (CCR1, CCR5) and by invading T cells (CCR2, CCR4, CXCR3) despite CCR5-deficient mice showing no difference in EAN severity [143–145]. Blocking of CXCR7 resulted in a significantly reduced EAN score, while blocking of CXCR4 lead to increased severity. This increase was accompanied by higher IFN-γ, IL-12, and TNF-α mRNA expression in regional lymph nodes and spleen as well as by increased serum levels of IFN-γ. The expression of ICAM-1 and VCAM-1 on vascular endothelial cells of the sciatic nerve was upregulated using CXCR4 blocking compounds which resulted in an increased infiltration of CD4^+^ T cells and macrophages to the sciatic nerve. Interestingly, blocking of both CXCR7 and CXCR4 lead to a significant disease suppression. These data suggest a functional hierarchy of these receptors [146].

Very Late Antigen-4 (VLA-4), a dimer of α4 and β1 integrin on leukocytes, plays an important role in pathogenic leukocyte trafficking in many chronic inflammatory diseases. A counterligand of α4 integrin is FNCS1 expressed by microvascular endothelial cells from CIDP patients in vitro and in situ. FNCS1-blocking antibodies inhibited leukocyte trafficking at the human BNB in vitro*.* Furthermore, these antibodies ameliorated the spontaneous neuritis in B7-2^−/−^ NOD mice. FNCS1 is also expressed in microvessels in nerve biopsies of CIDP patients arguing for a potential role in leukocyte trafficking at the BNB in CIDP [147].

Targeting leukocyte trafficking across the BNB could be a promising therapeutic strategy in inflammatory neuropathies. Statins, for example, inhibit Rho-mediated transendothelial T cell migration and T cell activation and proliferation [148, 149]. In EAN, statins ameliorated clinical symptoms and nerve inflammation by decreasing the number of Th1 and Th17 cells in sciatic nerves and increasing the number of T_regs_ [116, 150]. In vitro experiments indicate that statins inhibit the TNF-α-mediated CCL2 release by endoneurial endothelial cells and reduce CD40 expression and thereby transendothelial migration [151–154].

Several studies using different statins showed that oral administration as well as multiple injections of statins are not optimal for efficacy. Therefore, a localized, controlled, and sustained release system based on statin-embedded nanoparticles was introduced [155]. Bilateral peri-neural administration of such a nanoparticle-based statin delivery system proofed to significantly attenuate EAN clinical severity and reduce peripheral nerve morphological and functional deficits [156]. Reducing migration of autoreactive leukocytes across the blood-nerve barrier using a peri-neural nanoparticle-based drug delivery platform might therefore be a future treatment option in localized nerve disorders.

To further decipher the mechanisms of leukocyte trafficking at the BNB, real-time in vivo models are needed. Using in vivo imaging techniques such as intravital microscopy or magnetic resonance neurography (MRN) (discussed in detail below) might help in observing the underlying mechanisms of peripheral nerve infiltration under physiological conditions.

## MR imaging and neurography

Visualizing disease activity and immune cell infiltration and monitoring the effect of anti-inflammatory treatments require insight into the PNS. Electrophysiology is the mainstay of diagnosing and monitoring neuritis, but it has limited spatial resolution and often poorly correlates with clinical impairments especially in cases with a high level of axonal injury. Nerve biopsy is no option for monitoring treatment success as it is an invasive and one-time diagnostic procedure. MRI of peripheral nerves, also termed MRN, is therefore an interesting tool for repeatedly assessing PNS integrity and immune cell infiltration in inflammatory neuropathies.

MRN allows directly visualizing damage in the nervous system and pinpointing the exact localization of nerve lesions even in clinically unaffected nerves [157]. Therefore, this technique may help to improve therapy decisions of anti-inflammatory agents and targeting cellular trafficking (reviewed in [158, 159]). In a multiple sclerosis animal model, infiltration of cells labeled with iron particles has been monitored by iron oxide-enhanced MRI [160]. In an experimental setting, injection of labeled inflammatory cells thus allows for visualizing lesion formation and disease activity. Macrophage invasion in animals treated with Fingolimod has also been monitored with this technique as well as nerve repair [161, 162]. In adoptive transfer EAN, infiltration of macrophages into spinal nerves leading to myelin damage was detected with MRI and precedes clinical onset. Based on these findings, blocking invasion mechanisms of macrophages reduces clinical disease in an animal model of inflammatory neuropathies [163]. In the same model, using gadofluorine M-enhanced MRN, nerve injury and recovery was observed [164]. Beside injecting labeled cells, a specific uptake of intravenous injected iron oxide nanoparticles by innate immune cells as well as brain-resident cells (e.g., activated microglia, infiltrating macrophages, neutrophils) allows for studying disease activity in vivo without targeting adaptive immune cells [165].

Overall, MRN and imaging allow for directly observing lesion formation and if using certain contrast enhancing agents even the infiltration of cells. This technique can thus improve diagnosis and treatment monitoring and might help improving the effect of novel therapeutic strategies in inflammatory neuropathies.

## Selected candidate therapeutic compounds

Plasma exchange and IVIg are effective in GBS while plasma exchange, IVIg, and glucocorticoids are the primary treatment options in CIDP and have been reviewed elsewhere [166, 167]. In addition to such established treatments, animal models of inflammatory neuropathies have enabled the preclinical testing of a multitude of potential novel therapeutic agents. Recently, several interesting new compounds have been introduced and may gain relevance in PNS autoimmunity.

### Fingolimod

Fingolimod (FTY720) is a fungus-derived orally bioavailable immunomodulatory drug that therapeutically targets the egress of lymphoid cells from secondary lymphoid organs. Fingolimod binds to sphingosine-1-phosphate receptor (S1P) thereby sequestering lymphocytes in lymphoid organs [168]. Potential neuroprotective effects have additionally been suspected [169]. Fingolimod is an effective treatment in experimental autoimmune encephalomyelitis [170–172], the animal model of multiple sclerosis and has been approved for the treatment of relapsing-remitting multiple sclerosis based on successful randomized controlled trials [173, 174]. Fingolimod treatment also protects from EAN [175] and ameliorates the spontaneous chronic inflammatory neuropathy in B7-2-deficient NOD mice [104, 117]. Fingolimod constitutes a promising therapeutic candidate especially in chronic inflammatory neuropathies. Recent data indicate Fingolimod to induce repair by cultured adult Schwann cells. Therefore, Fingolimod might enhance regeneration of the PNS [176]. Furthermore, Fingolimod was shown to decrease the pro-inflammatory capabilities of antigen-presenting cells (APCs) and therefore reduce T cell activation, proliferation and differentiation [177]. A phase-III clinical trial, however, was aborted due to lack of a significant benefit of Fingolimod vs. placebo [178].

### Rituximab/Ocrelizumab/Ofatumumab

Rituximab is a chimeric murine/human monoclonal antibody binding CD20, that is expressed on the surface of B cells of most developmental stages [179]. Rituximab is approved for the treatment of certain lymphomas and leukemia and is used off-label in some autoimmune disorders [180, 181]. Several reports describe efficacy of rituximab in CIDP [158], and B cell-directed treatment has been reported in the B7-2-deficient NOD animal model of CIDP [103]. Another anti-CD20 antibody is the humanized antibody Ocrelizumab (reviewed in [182]). A phase II, randomized, placebo-controlled multicentre trial indicated Ocrelizumab to be a promising therapeutic antibody for relapsing-remitting multiple sclerosis [183]. Three phase III clinical trials found Ocrelizumab to be efficacious in both relapsing-remitting multiple sclerosis (RRMS) and primary progressive multiple sclerosis (PPMS) [184, 185]. Clinical development of Ocrelizumab for the treatment of PPMS and RRMS is currently ongoing. Ofatumumab, a human monoclonal antibody binds to a distinct CD20 epitope. It is approved for chronic lymphocytic leukemia and tested for RRMS. A phase II trial indicated that Ofatumumab reduces the number of MRI lesions in multiple sclerosis [186]. A phase III trial in multiple sclerosis is being planned. Summarizing, the data suggest that treatments targeting CD20^+^ B cells are effective in multiple sclerosis and therefore also constitute interesting pharmaceutical candidates in inflammatory neuropathies.

### Quinpramine

A novel group of chimeric compounds derived from the tricyclic antidepressant Imipramine and the malaria drug Quinacrine demonstrated antiprion effects [187], resulting from intracellular cholesterol redistribution [188]. Anti-inflammatory effects have been demonstrated in an animal model of human multiple sclerosis [189]. In EAN, Quinpramine ameliorated the clinical and histological severity with reduced immune cell infiltration and inflammatory myelin destruction in the PNS. In cell culture studies Quinpramine took effect on antigen-presenting cells by reducing their MHC class II surface expression and cell surface localization of cholesterol [190]. Thus, by redistributing cholesterol-rich membrane domains to intracellular compartments, Quinpramine may reduce cell surface availability of MHC class II and therefore inhibit autoimmune activation [187]. The anti-inflammatory compound Quinpramine may thus constitute a potential future therapeutic option in GBS.

### Alemtuzumab

Alemtuzumab is a humanized monoclonal antibody against CD52 expressed on the surface of lymphocytes, monocytes, and macrophages. Application of this antibody leads to complement-dependent cell lysis, antibody-dependent cytotoxicity, and apoptosis which results in a 12-month-lasting lymphopenia. Alemtuzumab has been approved for treating multiple sclerosis and leukemia [191]. In IVIG-dependent CIDP, Alemtuzumab prolonged remission in young patients with short disease duration [192, 193]. Another study reported successful treatment of a 79-year-old patient with overlapping features of Miller Fisher syndrome and Bickerstaff brainstem encephalitis with Alemtuzumab [194]. Despite these promising results, potential autoimmune adverse effects such as autoimmune thyroiditis and other immunologic effects may limit the applicability of the drug [192, 195].

### Etanercept

Etanercept is a dimeric, recombinant human tumor necrosis factor (TNF) receptor, fused to human IgG1 capable of binding TNF-α. It is used effectively as an antagonist of TNF-α in rheumatoid and psoriatic arthritis by reducing inflammation and pain [196]. In a study with 10 CIDP patients, three patients showed significant improvement, three other possible improvement, one complete resolution of weakness, one successfully discontinued prednisone, and one temporarily relapsed after treatment ended [197]. Etanercept as an anti-TNF-α drug may be a future treatment option for patients not responding to standard therapies. On the other hand, Etanercept treatment has been reported to trigger multiple sclerosis-like autoimmunity with demyelination of the central nervous system [198].

### Complement inhibitors and modulators (e.g., Eculizumab)

Different lines of evidence support the role of complement in the pathogenesis of subtypes of human GBS. Complement deposition can be visualized on either Schwann cells or axonal membranes [199]. Anti-ganglioside antibodies can activate complement and cause complement-mediated disruption of ion channels and other nodal structures [200, 201]. Complement inhibitors and modulators ameliorate axonal injury in murine GBS models [48, 202]. Previous studies successfully demonstrated the principle of complement inhibition in GBS models through soluble complement receptors [203], complement depletion by cobra venom factor [204], and the complement inhibitor APT070 [205], although EAN severity was not reduced in complement component C6-deficient rats [206]. The monoclonal antibody Eculizumab for example is directed against human complement component C5 and is approved for treating paroxysmal nocturnal haemoglobinuria, in which attacks of complement-dependent haemolysis occur. This biological was effective in a murine axonal GBS model and appeared to be partially efficacious in a patient with severe GBS [48]. Therefore, Eculizumab is currently being investigated in patients with GBS in Japan (ClinicalTrials.gov Identifier: NCT02493725) and in a monocentric study in Scotland (ClinicalTrials.gov Identifier: NCT02029378). Therefore, complement-directed treatments constitute promising candidates for future GBS treatments and clinical trials are ongoing.

## Conclusions

Our understanding of disease mechanisms in inflammatory neuropathies has improved in recent years. This is in part due to the introduction of novel animal models and development of new immunological concepts. In addition, new diagnostic techniques such as MRN and experimental new treatments are increasingly applied to patients who fail to respond to established therapies. Despite such progress, the following open questions remain to be addressed in the future.

Although several candidate genes conferring disease susceptibility to GBS have been reported, it remains a central question why some, but not other patients, develop GBS after exposure to a certain pathogen. Little is known about determinants of disease susceptibility in inflammatory neuropathies, and predicting or even influencing the risk of disease in GBS-prone patients would have high clinical relevance. Non-hypothesis-driven genetic susceptibility approaches—such as genome-wide association studies and TCR sequencing—could help to predict the risk of GBS and identify self-reactive T cell clones and antigens if sufficient patient numbers are collected despite the rareness of the disease [207].

Furthermore, molecular determinants of disease severity could add to established clinical predictors [1, 7]. It also remains to be addressed why PNS autoimmunity is monophasic in some but relapsing or chronic progressive in other patients. Animal models are genetically and immunologically far more homogenous than human patients and thus allow to address this aspect more specifically. If targeted inventions could “chronify” acute animal models (as described for CsA above), this could identify determinants of disease course in inflammatory neuropathies. As previously discussed in detail [8], converting experimental, animal-based science into clinical relevance appears as an insurmountable obstacle. Solving the bench-to-bedside transfer problem thus remains the most relevant challenge in inflammatory neuropathies.

## Abbreviations

AIDP: Acute inflammatory demyelinating polyneuropathy
AMAN: Acute motor axonal neuropathy
AMSAN: Acute motor and sensory axonal neuropathy
APC: Antigen-presenting cell
AT-EAN: Adoptive transfer experimental allergic neuritis
Breg: Regulatory B cell
CDR: Primary structure and complementarity-determining region
CFA: Complete Freund’s adjuvant
CIAP: Chronic idiopathic axonal polyneuropathy
CIDP: Chronic inflammatory demyelinating polyradiculoneuropathy
CNS: Central nervous system
CsA: Cyclosporine A
CSF: Cerebrospinal fluid
EAE: Experimental autoimmune encephalomyelitis
EAN: Experimental allergic neuritis
FNCS1: Fibronectin connecting segment-1
Fox: Forkhead box
GBS: Guillain-Barré syndrome
ICAM: Intercellular adhesion molecule
ICOS: Inducible costimulator
IdeS: Immunoglobulin G-degrading enzyme of *Streptococcus pyogenes*
IFN: Interferon
Ig: Immunoglobulin
IL: Interleukin
IPEX: Immunodysregulation polyendocrinopathy enteropathy X-linked syndrome
IVIg: Intravenous immunoglobulin
MBP: Myelin basic protein
MFS: Miller Fisher syndrome
MHC: Major histocompatibility complex
MMN: Multifocal motor neuropathy
Mpz: Myelin protein zero
MRI: Magnet resonance imaging
MRN: Magnet resonance neurography
Nav: Voltage-gated sodium channel
NF: Neurofascin
NOD: Non-obese diabetic
PD: Programmed death
PDN: Painful diabetic neuropathy
PMP: Peripheral myelin protein
PNS: Peripheral nervous system
PPMS: Primary progressive multiple sclerosis
RRMS: Relapsing-remitting multiple sclerosis
S1P: Sphingosine-1-phosphate
TCR: T cell receptor
Teff: Effector T cell
Th: T helper cell
TNF: Tumor necrosis factor
Treg: Regulatory T cell
VLA-4: Very Late Antigen
VMD: Vitelliform macular dystrophy
ZIKV: Zika virus
